# The novel regulator HdrR controls the transcription of the heterodisulfide reductase operon *hdrBCA* in *Methanosarcina barkeri*

**DOI:** 10.1128/aem.00691-24

**Published:** 2024-05-29

**Authors:** Sicheng Zhang, Yi Chen, Shuxin Wang, Qing Yang, Huan Leng, Pengyan Zhao, Leizhou Guo, Lirong Dai, Liping Bai, Guihong Cha

**Affiliations:** 1 Key Laboratory of Development and Application of Rural Renewable Energy, Biogas Institute of Ministry of Agriculture and Rural Affairs, Chengdu, China; 2 Terra Research and Teaching Centre, Microbial Processes and Interactions (MiPI), Gembloux Agro-Bio Tech, University of Liège, Gembloux, Belgium; University of Illinois Urbana-Champaign, Urbana, Illinois, USA

**Keywords:** *Methanosarcina barkeri*, substrate metabolism, methane, methanogens, regulator

## Abstract

Methanogenic archaea play a key role in the global carbon cycle because these microorganisms remineralize organic compounds in various anaerobic environments. The microorganism *Methanosarcina barkeri* is a metabolically versatile methanogen, which can utilize acetate, methanol, and H_2_/CO_2_ to synthesize methane. However, the regulatory mechanisms underlying methanogenesis for different substrates remain unknown. In this study, RNA-seq analysis was used to investigate *M. barkeri* growth and gene transcription under different substrate regimes. According to the results, *M. barkeri* showed the best growth under methanol, followed by H_2_/CO_2_ and acetate, and these findings corresponded well with the observed variations in genes transcription abundance for different substrates. In addition, we identified a novel regulator, MSBRM_RS03855 (designated as HdrR), which specifically activates the transcription of the heterodisulfide reductase *hdrBCA* operon in *M. barkeri*. HdrR was able to bind to the *hdrBCA* operon promoter to regulate transcription. Furthermore, the structural model analyses revealed a helix-turn-helix domain, which is likely involved in DNA binding. Taken together, HdrR serves as a model to reveal how certain regulatory factors control the expression of key enzymes in the methanogenic pathway.

**IMPORTANCE:**

The microorganism *Methanosarcina barkeri* has a pivotal role in the global carbon cycle and contributes to global temperature homeostasis. The consequences of biological methanogenesis are far-reaching, including impacts on atmospheric methane and CO_2_ concentrations, agriculture, energy production, waste treatment, and human health. As such, reducing methane emissions is crucial to meeting set climate goals. The methanogenic activity of certain microorganisms can be drastically reduced by inhibiting the transcription of the *hdrBCA* operon, which encodes heterodisulfide reductases. Here, we provide novel insight into the mechanisms regulating *hdrBCA* operon transcription in the model methanogen *M. barkeri*. The results clarified that HdrR serves as a regulator of heterodisulfide reductase *hdrBCA* operon transcription during methanogenesis, which expands our understanding of the unique regulatory mechanisms that govern methanogenesis. The findings presented in this study can further our understanding of how genetic regulation can effectively reduce the methane emissions caused by methanogens.

## INTRODUCTION

Methanogenic archaea are single-celled organisms, which were first discovered about a century ago (1, 2). Recently, methanogens have received increased research interest because of a key role in climate change via methane emissions, which represent a greenhouse gas (3). These unusual microorganisms grow using a limited set of substrates and obtain energy from methanogenic processes (3). Most methanogens only use H_2_/CO_2_ or formate as a growth substrate, while members of the *Methanosarcina* genus show greater metabolic diversity, for example, certain species can utilize H_2_/CO_2_, simple carbon (C1-C2) compounds (methanol, methylamines, dimethylamines, trimethylamines, methanethiol, and methyl-sulfides), and acetate as growth substrates (4).

The *Methanosarcina* species that utilize H_2_/CO_2_ as a substrate acquire electrons from H_2_ via two types of hydrogenases, namely, membrane hydrogenase Ech and the F_420_-dependent hydrogenases Frh/Fre (5). These electrons are ultimately used in the reduction of CO_2_ to methane (6). Under aceticlastic methanogenesis, the substrate acetate is initially activated to acetyl-CoA (7). Subsequently, the carbonyl group of acetyl-CoA is oxidized to CO_2_, while the methyl group is transferred to tetrahydromethanosarcinopterin (H_4_SPT), which produces methyl-tetrahydromethanosarcinopterin (methyl-H_4_SPT). In the methylotrophic pathway, methyl groups from C1 compounds, such as methanol or methylamine, are directly transferred to coenzyme M, which forms methyl-CoM (8). This central metabolic intermediate is then reduced to methane (9). Multiple types of substrate-dependent methyl-CoM transferase complexes have already been described in methanogens (10).

As some *Methanosarcina* species can utilize different substrates to obtain the energy necessary to grow, it would be plausible that distinct regulatory mechanisms can either stimulate or inhibit the transcription of key enzymes of different methanogenic pathways when the substrate changes (11, 12). For instance, in *Methanosarcina acetivorans*, MsrA, and MsrB can activate the *mtaCB1* operon, whereas both MsrD and MsrE can activate the *mtaCB2* operon and repress the *mtaCB3* operon (13). These regulators control the expression of methanol methyltransferase isozymes in *M. acetivorans* (13). In addition, it was reported that MreA can regulate various methanogenic pathways in *M. acetivorans* (14, 15). For example, MreA is involved in the regulation of genes related to acetate activation, for example, *pta*, *cdh*-1, and *ack* (Aceticlastic pathway), as well as genes, which encode methyltransferases such as *mtbA*, *mtmCB*, *mtbCB*, *mttCB*, *mtaA*, and *mtaCB* (methylotrophic pathways) (14).

Different forms of another key enzyme, heterodisulfide reductase (Hdr), also exist for different substrate conditions. Biochemical studies have revealed two distinct classes of Coenzyme B-Coenzyme M heterodisulfide (CoB-S-S-CoM) reductase, a key enzyme required for anaerobic respiration in methane-producing archaea (16). Hydrogenotrophic methanogens, for example, *Methanobacterium thermoautotrophicum,* use the cytoplasmic complex HdrBCA to reduce heterodisulfide, while Methanosarcinales produce membrane HdrDE for the same function; in this case, methanophenazine serves as the electron carrier (5). However, all five of the Methanosarcinales genomes that have been sequenced thus far (*Methanosarcina barkeri* Fusaro, *M. acetivorans* C2A, *Methanosarcina mazei*, *Methanosaeta thermophila*, *Methanococcoides burtonii*) were found to contain both *hdrDE* as well as the putative *hdrBCA* operon (16–18). The *M. acetivorans* HdrABC appears to be specifically involved in methylotrophic methanogenesis, based on reduced growth and methanogenesis rates of an *hdrBCA* mutant on methylotrophic substrates and downregulation of the genes transcription during growth on acetate (16). However, very little is known about the regulation of *hdrBCA* genes under different substrate regimes. In this study, the regulatory mechanisms underlying gene transcription in *M. barkeri* were investigated under substrate regimes of acetate, H_2_/CO_2_, and methanol. We have reported a novel regulator HdrR of *M. barkeri*, specifically activating transcription of the operon encoding HdrB, HdrC, and HdrA. Genetic, biochemical, and structural model analyses demonstrated how this protein not only binds to the *hdrBCA* promoter region but also affects heterodisulfide (CoB-S-S-CoM) reductase core gene *hdrBCA* transcription and the subsequent methane emission behavior.

## MATERIALS AND METHODS

### Media and growth conditions

The *M. barkeri,* strain DSM 800, was obtained from DSMZ (Braunschweig, Germany). The microorganism was cultured in 120 mL serum bottles with a working volume of 30 mL. The growth medium consisted of mineral salt medium and either H_2_/CO_2_, acetate, or methanol. For the H_2_/CO_2_ substrate group, the 50 mL of growth medium contained only the mineral salts medium, while the headspace of the serum bottle was filled with a gas mixture of 80% (v/v) H_2_ and 20% (v/v) CO_2_. For the acetate and methanol substrate groups, the headspace of the serum bottle was filled with a gas mixture of 80% (v/v) N_2_ and 20% (v/v) CO_2_, while the 30 mL medium consisted of the mineral salt medium plus sodium acetate (50 mM) or methanol (50 mM), respectively. A solution of cysteine and Na_2_S·9H_2_O [each at a concentration of 2.5%) was added (1% (v/v)] to the medium to create the conditions necessary for substrate reduction. The final pH of the medium was adjusted to 7.4 with NaHCO_3_. The bottles were then autoclaved at 121°C for 20 min, after which the medium reservoir bottles were cooled to room temperature. A 10 mL inoculum of *M. barkeri* in the mid-exponential phase (determined by monitoring CH_4_ production rate) was added to the medium. The microorganisms were then cultured under three substrate regimes, with each treatment group subjected to anaerobic conditions at 37°C. Before the initiation of the continuous cultivation experiments, *M. barkeri* was allowed to adapt to the respective substrate through three successive passages. Different culture times corresponding to specific treatments were determined based on the results from pre-experiments.

### Growth curves and chemical analyses

The *M. barkeri* microorganisms grown in the presence of either H_2_/CO_2_, acetate, or methanol were maintained at 37°C at all times. After adaptation to the respective substrate, that is, approximately 30 generations, the cultures were diluted by a factor of 10^4^ into 25 mL Balch tubes containing 10 mL of medium. The cultures were incubated in quadruplicates at a steady temperature of 37°C. Growth was measured every 12 or 24 h by measuring the optical density at 600 nm (OD_600_) with a Spectronic 21 spectrophotometer (Milton Roy, Ivyland, PA, USA).

A pressure lock syringe (Vici, Schenkon, Switzerland) was used to take gas samples (200 µL) from the headspace of the vials or serum bottles at different time points. CH_4_ concentrations were measured using an Agilent GC7820A gas chromatography system (Agilent, Santa Clara, CA, USA) equipped with a Porapak Q column (Waters Corporation, Milford, MA, USA; length, 3 m; inner diameter, 0.32 mm) and a thermal conductivity detector. The column, oven and detector temperatures were set to 65, 120, and 130°C, respectively. The carrier phase gas was Argon with a flow rate of 27 mL min^−1^. The gas pressure in the headspace of vials or serum bottles was determined using a barometer at room temperature (Ashcroft, Stratford, CT, USA). The total amount of gas products was calculated based on Avogadro’s law after calibration with gas mixture (Standard gas mixture, N_2_ : CH_4_ : C_2_H_6_ : C_3_H_8_ : iso-C_4_H_10_ : iso-C_5_H_12_ = 61.41% : 14.24% : 8.21% : 10.02% : 3.80% : 2.32%). The concentrations of CH_4_ were calculated based on an external calibration curve.

### RNA extraction, RNA-seq, and quantitative real-time PCR (qRT-PCR)

Cells representing the mid-exponential phase were collected from all treatments for total RNA extraction, which was performed using a RNAprep pure Cell/Bacteria Kit (Cat. No. DP430; TIANGEN, Beijing, China). RNA purity and concentrations in the samples were determined by gel electrophoresis and a NanoDrop2000 spectrophotometer (Thermo Scientific, Waltham, MA, USA). Ribosomal RNA was removed from total RNA using the RiboMinus TM kit (Lot. No. 1539791, Invitrogen, Waltham, MA, USA). Samples were then submitted to GENEWIZ (Suzhou, China) for library construction and sequencing (19) on an Illumina HiSeq 4000 system (Illumina, San Diego, CA, USA). The sequencing reads were mapped to the *M. barkeri* DSM800 genome using Bowtie 2 (20). HTSeq 2.0 was used to determine the read numbers mapped to each gene, after which the fragments per kilobase per million reads (FPKM) for each gene could be calculated (21). Differential transcription analysis for the substrate conditions (acetate vs. CO_2_/H_2_, acetate vs. methanol, and methanol vs. CO_2_/H_2_) was performed using the DESeq2 (1.42.0) R package (22). Genes with an adjusted *P*-value < 0.01 from the DESeq2 package and a FPKM value > 100 in the groups sample were classified as demonstrating differential transcription.

For the qRT-PCR analyses, total RNA was extracted as described above; this step was followed by DNase I (Bio-Rad, USA) treatment. The RNA was then transcribed using the iScript cDNA Synthesis Kit (Bio-Rad, USA). Transcript levels were determined with the SsoAdvanced Universal SYBR Green Supermix (Bio-Rad) and a CFX96 Connect Real-Time PCR Detection System (Bio-Rad). The following cycling parameters were used: 95°C for 30 s followed by 40 cycles of 94°C for 15 s and 56°C for 30 s. After the main program, melt curve analysis was performed from 65 to 95°C, with an increment of 0.5°C and 0.5 s plate reading at each step. The sample DNA was diluted about 100 times when necessary. A positive control, in which only chromosomal DNA was added to the qPCR, and a negative control, in which only RNA without reverse transcriptase was used for the qPCR, were run under identical PCR amplification conditions. The abundance of the 16S rRNA was used as an internal standard for the standardization of results. The relative transcription levels of target genes were calculated using the Quantitation-Comparative CT(2^−ΔΔCT^) method (23).

### Data analysis

The FPKM (Fragments Per Kilobase of exon model per Million mapped fragments) value for each gene was calculated by normalizing the sequence coverage over the gene length and the read count mapped to the gene. The annotation of protein-coding genes in *M. barkeri* was performed based on the archaeal clusters of orthologous genes (24) (arCOG: ftp://ftp.ncbi.nih.gov/pub/wolf/COGs/arCOG/). The functions of analyzed genes mRNA were determined according to the “different hierarchies of function” category available in arCOG. The enrichment analyses of transcribed genes and pathways were performed using the DEseq2 package (22) and Fisher’s exact test, respectively, in R 4.4.0 version (25). The *P*-value was adjusted using the Benjamini and Hochberg methods (26), with a value of 0.01 set as the threshold for statistical significance. The general changes in gene expressions across different treatments were evaluated by permutational multivariate analysis of variance using the function Adonis in the vegan package, along with principal coordinates analysis (PCA) using the function *pca* in the *ape* package; these analyses were conducted in R, and applied the Bray-Curtis dissimilarity distance (27). The normality and homoscedasticity of data were evaluated using the software SPSS 21 (IBM, Armonk, USA). Pearson’s and Spearman’s correlation analyses, along with the Mantel Test and Partial Mantel Test (both of which were based on Spearman’s method), were performed in R with the vegan package. The null model method was used to assess deterministic versus stochastic processes of gene expression by keeping alpha and gamma diversity constant and calculating the Bray-Curtis dissimilarity distance (27), and the pipeline for this analysis is available at http://ieg.ou.edu/microarray/.

### Identification and analysis of protein HdrR

The genes encoding all HdrR in the selected genomes (Information on all the selected genomes (8028 in total) was downloaded from the NCBI genome database, please see Table S5) were identified by BLAST 2.12.0 (28) against local genomes, and hits with an e-value of <e^−3^ were assigned as candidates. The protein sequences of the MSBRM_RS03855 (HdrR) from *M. barkeri* DSM 800 were selected as query sequences to perform the BLASTP analysis. All the candidates were reexamined for their domain organization by searching the SMART database (29). All HdrR homologous proteins are listed in Table S6.

### For the phylogenetic analysis of HdrR

The HdrR protein sequence from *M. barkeri* DSM800 and HdrR homologous sequences (Table S6) from publicly available archaea genomes or meta-genomes were aligned using MAFFT version 7.0 (30). The maximum-likelihood Poisson correction model was generated using MEGA X (31). Bootstrap values were obtained using 1,000 replications. The initial tree for the heuristic search was automatically generated. The Neighbor-Join and BioNJ algorithms were applied to a pairwise distances matrix computed using the JTT model. Trees were visualized using the iTol webserver (32).

### Construction, verification, and complementation of mutant strains

*M. barkeri* knock-out mutant strains were constructed as previously described (33, 34). Briefly, flanking regions of the selected open reading frames (ORFs) were amplified via PCR, after which a neomycin resistance cassette was inserted in the middle; the entire element was then cloned into a pBluescript II SK plasmid through the Gibson assembly protocol (35). The resulting plasmids were used to integrate the mutated alleles into *M. barkeri* chromosomes by liposome-mediated transformation and allelic recombination. Notably, individual gene deletions removed most of the coding sequence, which resulted in an in-frame fusion peptide consisting of the first and last 10 amino acids to create non-polar mutations. In other words, the cassette does not include either a promoter or a terminator, with translational stop codons in all of the reading frames preceding the 5′-end of the ribosome binding site (RBS) of the *neo* encoding sequence, and another RBS at the 3′-end to overcome translational coupling of the downstream *hdrB* gene. The plasmid used for complementation was constructed using pJK027A, which–in the strain used in this study–expresses the genes under investigation via the strong, constitutive *P_mcrB-tetO_* promoter (33). The complementation plasmid was integrated into chromosomes of the respective knock-out mutant as a single copy via a site-specific recombination method described by Metcalf et al. (33). The primers and plasmids used to generate the mutants are listed in Tables S7 and S8**,** respectively.

### Bacterial two-hybrid (BACTH) assay

The bacterial two-hybrid (BTH) assays were performed as described (36). The pKNT25 and pUT18C plasmids carrying different target cloned inserts were co-transformed into *Escherichia coli* BTH101 competent cells. Co-transformed cells were plated on LB plates supplemented with ampicillin, kanamycin, isopropyl thio-D-galactopyranoside (IPTG), and X-gal at final concentrations of 100 µg/mL, 50 µg/mL, 0.5 mM, and 50 µg/mL, respectively. The corrected cells were cultivated at 30°C for 12–24 h. The pKNT25-zip and pUT18C-zip plasmids were used as positive controls (Euromedex), and the pKNT25 (without insert) and pUT18C-zip (fused with a leucine zipper protein) plasmids were used as negative controls. The BACTH assay primers and plasmids are listed in Tables S7 and S8**,** respectively.

### Construction of *hdrBCA* promoter reporter strains and β-galactosidase assay

To construct a *lacZ* reporter strain for measuring *hdrBCA* promoter activity in *M. barkeri* wild-type and mutant strains, a pCH003 plasmid was generated to transfer the transcriptional fusion of the *hdrBCA* promoter and the *lacZ* gene into *M. barkeri* strains. More specifically, the ~1 kb upstream sequence preceding the start codon of the *hdrB* gene, along with the *hdrB* encoding sequence with the RBS, from *M. barkeri* were amplified via PCR and cloned into a pBluescript II SK plasmid by the Gibson assembly method (35). Next, the following elements were added to the plasmid via Gibson assembly: a PCR fragment of the promoter-less *lacZ*-encoding gene (from the plasmid pMEV2) and a streptothricin acetyltransferase (*sat*) resistance gene cassette (from the plasmid pCH009) with a constitutive promoter right after the *hdrB* upstream sequence. The resulting pCH004 plasmid, which now contained the *hdrB* upstream promoter fused with the *lacZ* gene, a streptothricin resistance cassette, and the *hdrB* gene (in this order) was introduced into the pBluescript II SK vector. This plasmid was then transformed into different *M. barkeri* strains for allelic recombination to generate the *hdrBCA* promoter reporter strain. Galactosidase activity in the *M. barkeri* strains was determined using a kinetic assay, described previously by (37, 38). Briefly, three colonies from each *M. barkeri* strain were cultured in 10 mL Hengate tubes until they reached the exponential phase, and cell density (OD_600_ = 0.6) was measured. One milliliter of the cultures was harvested at 4°C by centrifugation at 8,000× *g*. Subsequently, cell pellets were resuspended and lysed by 200 µL of 20 mM Z-buffer, followed by shaking for 12 h at 37°C. Cell extracts were then centrifuged at 8,000× *g* and 30°C. One drop of SDS 0.01% and two drops of chloroform are added, and the Hengate tubes are mixed thoroughly for 10 s to permeabilize the cells. After letting chloroform settle down at the bottom of the tube, aliquots of 50 µL are transferred into a Synergy H4 Hybrid Multi-Mode Microplate Reader (BioTek Instruments) microplate containing 150 µL of Z buffer. Then, 40 µL of ONPG 0.4% are dispensed and the enzymatic reaction is carried out at 37°C for 30 min with a measurement of OD_420_ nm every 2 min in the microplate reader. The relative b-galactosidase activity in each of the 96 samples is then calculated by simple Excel file manipulation. It corresponds to [(OD_420nm_ at time *t*_2_ – OD_420nm_ at time *t*_1_)/*t*_2_ – *t*_1_ (min)]/OD_600nm_. The *t*_2_ and *t*_1_ time points are chosen to be located in the linear part of the kinetic.

### Protein expression and purification

The coding regions of *hdrR* were amplified via PCR and introduced into the expression vector pET-28a by Gibson assembly (35). The recombinant plasmid pET28a-*hdrR* was transformed into *E. coli* BL21 by heat shock at 42°C. For protein expression, 5 mL of an overnight culture of the *E. coli* strain harboring the appropriate plasmid was transferred to 500 mL of LB medium with 50 µg/mL kanamycin, and grown until OD_600_ reached a value between 0.4 and 0.6. Following the addition of IPTG to a final concentration of 0.1 mM, the cultures were further incubated in a shaker at 16°C for 15 h. Bacterial cells were harvested by spinning at 5,000 rpm, followed by resuspension in PBS buffer and lysis by JN-Mini ultra-high-pressure continuous flow cell disrupters (JNBIO, Guangzhou, China). The soluble fractions were collected by centrifugation at 15,000 *g* for 30 min at 4°C. His-tagged proteins were purified with Ni-NTA Agarose (Qiagen, Hilden, Germany) following the manufacturer’s instructions. The fraction was dissolved in 20 mL binding buffer (20 mM Na_2_HPO_4_, 0.5 M NaCl, and 20 mM Imidazole, pH 7.4) and centrifuged, and then the supernatant was filtered through a 0.45 µm filter and applied to a His Bind Column equilibrated with the washing buffer (20 mM Na_2_HPO_4_, 0.5 M NaCl, and 50 mM Imidazole, pH 7.4). After elution of the proteins lacking the His tag using washing buffer, the recombinant protein was eluted using elution buffer (20 mM Na_2_HPO_4_, 0.5 M NaCl, and 250 mM Imidazole, pH 7.4). All the recombinant proteins were loaded onto the PD-10 Desalting column containing Sephadex G-25Medium (Amersham Biosciences, USA) to remove the salt. The purified recombinant HdrR was analyzed by SDS–PAGE, and the Bradford method was employed to calculate protein concentrations (39).

### Electrophoretic mobility shift assay (EMSA)

The promoter region of the *hdrBCA* operon (111 bp) was amplified via PCR and gel purified, after which the sequence was labelled with biotin using a Biotin 3′-end DNA labelling Kit (Thermo Scientific, USA). For the binding reactions, a DNA probe (20 mM) was added to different amounts of purified 6× His HdrR protein in a 20 µL system containing 2 µL 10× Binding buffer, 1 µL 50% Glycerol, 1 µL 1% NP40, 1 µL 1M KCl, 1 µL 100 mM MgCl2 and 0.25 µL 1 µg/µL Poly (dI : dC). An excess amount of unlabeled target DNA was added to one of the samples to compete for protein binding; this sample served as the control. In addition, bovine serum albumin (BSA)–which does not bind to target DNA–was added to one of the reactions as a negative control. The binding reactions were incubated at 25°C for 2 h and loaded onto a 6% DNA retardation gel at 110 V in 0.5× TBE [45 mM Tris (pH 8.3), 45 mM boric acid and 1 mM EDTA] and subjected to electrophoresis at 4°C for 60 min. The DNA probe was transferred to a nylon membrane (Bio-RAD, USA) at 400 mA for 30 min and subsequently UV-crosslinked at 302 nm for 20 min. Stabilized streptavidin-HRP (50 µL) was added and the mixture was incubated at room temperature for 30 min in a Blocking Buffer. After washing with 1× Wash Buffer three times, the chemiluminescent signal was detected using Luminol/Enhancer Solution and Nucleic Acid Detection Blocking Buffer according to the manufacturer’s protocol.

### DNase I footprinting assay

The DNase I footprinting assays were performed as described (40). The promoter sequence of the *hdrBCA* operon (111 bp) was amplified via PCR using forward primers modified with FAM. The FAM-labeled probes were gel-purified and quantified with a NanoDrop spectrometer (Thermo Scientific). For the DNase I footprinting assay, 20 mM of probes were incubated with 50 nM of 6× His HdrR in 40 µL of buffer containing 10 mM HEPES (pH 7.3), 25 mM KCl, 2.5 mM MgCl2, 0.5% NP-40, 50 ng/µL poly(dI·dC), and 2.5% glycerol. After incubation for 30 min at 25°C, 10 µL of solution containing approximately 0.05 unit of DNase I (NEB) was added to the mixture. The reaction was stopped by adding 200 mM of EDTA. DNA samples were then extracted with phenol/chloroform and precipitated with ethanol, after which the pellets were dissolved in 30 µL of RNase/DNase-free water. The samples were run through a 3730 DNA Analyzer, and the results were viewed using the Peak Scanner feature (Applied Biosystems, Waltham, MA, USA).

### AlphaFold2 prediction and docking analysis

The amino acid sequence of HdrR was determined based on the *M. barkeri* genome. Computational homology modelling was performed to identify novel HdrR regulators. The three-dimensional structure for the putative HdrR sequence was built using AlphaFold2 (41). The energy-minimization molecule models included in UCSF Chimera (42) (version 1.12) were used to minimize the energy for the structures from the previous step. The ligand structures were drawn, a charge was added and the structures were transformed to 3D conformations. Next, potential ligands, which were modelled based on DNA sequence, were docked to the HdrR using AutoDock VinA (43) (version 1.1.2). Receptor and ligand options in AutoDock Vina were set as default. The grid box in the docking procedure was defined to include the helix-turn-helix (HTH) motif, and corresponding residues appeared in the binding site of crystal structures for homologous proteins. Receptor and ligand options in AutoDock Vina were set as default. The number of binding modes, exhaustiveness of search, and maximum energy difference (kcal mol^–1^) parameter were set as 9, 8, and 3, respectively. All of the structures were visualized, and exported as images using PyMOL (http://www.pymol.org).

### Transformation methods

*E coli* strains were transformed by heat shock at 42°C. Liposome-mediated transformation was used for *M. barkeri* as described (44). Briefly, for liposome-mediated transformation, cells from log-phase cultures (OD_600_ between 0.4 and 0.6) were collected by centrifugation and resuspended in 0.85 M sucrose at a density of about 10^9^ cells per milliliter. DNA: liposome complexes were formed by mixing 10 µL DOTAP (Boehringer Mannheim) in 100 µL of 20 mM Hepes (pH 7.6) with 2.5 µg of plasmid DNA in 50 µL of 20 mM Hepes (pH 7.6), followed by a 15 min incubation at room temperature. A 1.0 mL portion of the resuspended cells was added to the DNA : liposome suspension and incubated for 6 h at room temperature. With these cell and DNA concentrations, the maximum transformation frequency of *M. barkeri* was achieved with 15 µL of DOTAP reagent. For all methods, cells were transferred to 10 mL of broth medium after transformation, incubated at 37°C for 16 h, and then plated on medium with antibiotic.

### *In silico* methods

Protein domain predictions were made using SMART (29) and BLAST (28). MAFFT version 7.0 (30) was used to determine sequence identity and form amino acid sequence alignments. The Identification of the promoter sequence of the *hdrBCA* from *M. barkeri* DSM 800 was selected as query sequences to perform the BLAST analysis. MAFFT version 7.0 was used to determine sequence identity and form promoter sequence alignments. Identification of the HdrR-protected region in *hdrBCA* promoter by DNase I footprinting assay was made, and sequence conservation of the identified HdrR-binding region in different archaea species containing HdrR homologs was performed. The sequence binding region was visualized using the WebLogo webserver (45).

## RESULTS

### Substrate acquisition by *M. barkeri* DSM800

**FIG 1:**
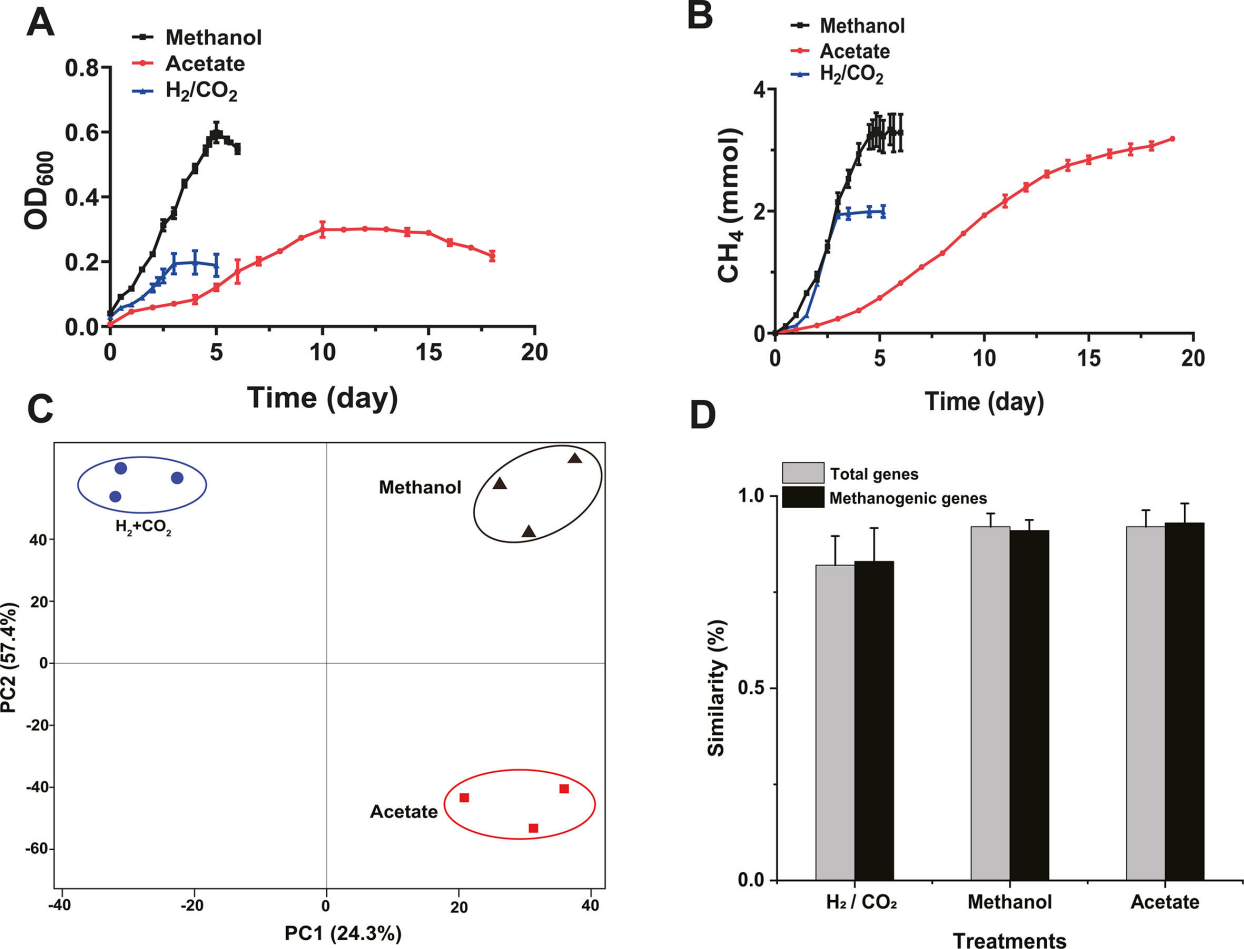
*M. barkeri* growth and gene transcription across different substrates. (A) Growth curves of *M. barkeri* for three distinct substrate regimes. (B) Accumulation of methane in the headspace of bottles representing different substrate treatments over an incubation time of 20 days. (C) PCA of total gene transcription, based on the arCOG database, across different treatments. (D) Measure of similarity (1-Bray-Curtis dissimilarity) among replicates in different treatments. All data are presented as means ± standard deviations.

The *M. barkeri* DSM800 strain exhibited distinct growth curves when cultivated on different substrates (Fig. 1A; Table 1). In all substrate groups, CH_4_ production increased continuously until the substrate was completely exhausted. However, the group supplied with methanol reached this point in the shortest time, followed by the groups supplied with H_2_/CO_2_ and acetate. This suggests that *M. barkeri* demonstrates the highest metabolic rate when grown with methanol as the substrate (Fig. 1A). Also, the microorganisms provided with methanol as a substrate showed the highest average CH_4_ production rate (*P* < 0.01), followed by the groups that received H_2_/CO_2_ and acetate as a substrate (Fig. 1B; Table 1). This result further supports the notion that microorganisms achieve the highest metabolic rate when methanol is the substrate.

**TABLE 1:** Growth of *M. barkeri* strains in various media^*a*^

Substrate	Generation time (h)^*b*^	Lag phase (h)^*c*^	Maximum growth (OD_600_)^*d*^
Methanol	29 ± 2.5	24 ± 1.5	0.601 ± 0.10
Acetate	65 ± 12.1	72 ± 3.0	0.312 ± 0.05
H_2_/CO_2_	52 ± 3.1	36 ± 4.0	0.201 ± 0.04

^*a*^ The substrates were added to the growth medium in the following amounts: methanol (500 mmol); acetate (500 mmol); and H_2_/CO_2_ (80%/20%) at 250 kPa.

^*b*^ The growth rate was measured by following the optical density of the cultures. The maximum optical density is reported in the table. The reported values are the mean ± standard deviation of triplicates.

^*c*^ Lag time is defined as the time required for the culture to reach half of the maximal OD_600_ value following a transfer to fresh media, with incubation at 37°C.

^*d*^ Maximum growth was quantified when the culture reached the maximal OD_600_ value after a transfer to fresh media, with incubation at 37°C.

### Overall changes in gene transcription

A null model method was employed to assess the deterministic versus stochastic transcription of total and methanogenic genes. The PCA results revealed significant variations (*P* < 0.001) in gene transcription in response to different substrates (Fig. 1C). Subsequently, the data, reflecting triplicate experiments for three different substrates, were subjected to PCA (Fig. 1C). The findings indicated that the substrate could explain 82% of the variation in gene transcription. Therefore, gene transcript abundance in *M. barkeri* is largely dependent on the carbon source. A similar observation was made for both total and methanogenic genes (Fig. 1D), suggesting that the substrate utilized by *M. barkeri* determines not only methanogenic dynamics but also all necessary cellular processes for survival.

### Comparing the abundance of gene transcripts in microorganisms grown under different substrate conditions

The gene transcription profiles for *M. barkeri* were observed to change according to the substrate under investigation. As per the arCOG database, the category of functional metabolism demonstrated the highest proportion (>40%) of differentially transcribed genes across groups (Table S1). This suggests that the primary concern of the observed differences in gene transcript abundance is metabolism. Differences in gene transcript abundance between substrate groups were shown in the clusters of “Amino acid transport and metabolism”, “Inorganic ion transport and metabolism”, “Energy production and conversion”, “Coenzyme transport and metabolism”, “Translation and ribosomal structure”, and “Biogenesis pathway” (Tables S2 to S4).

**FIG 2:**
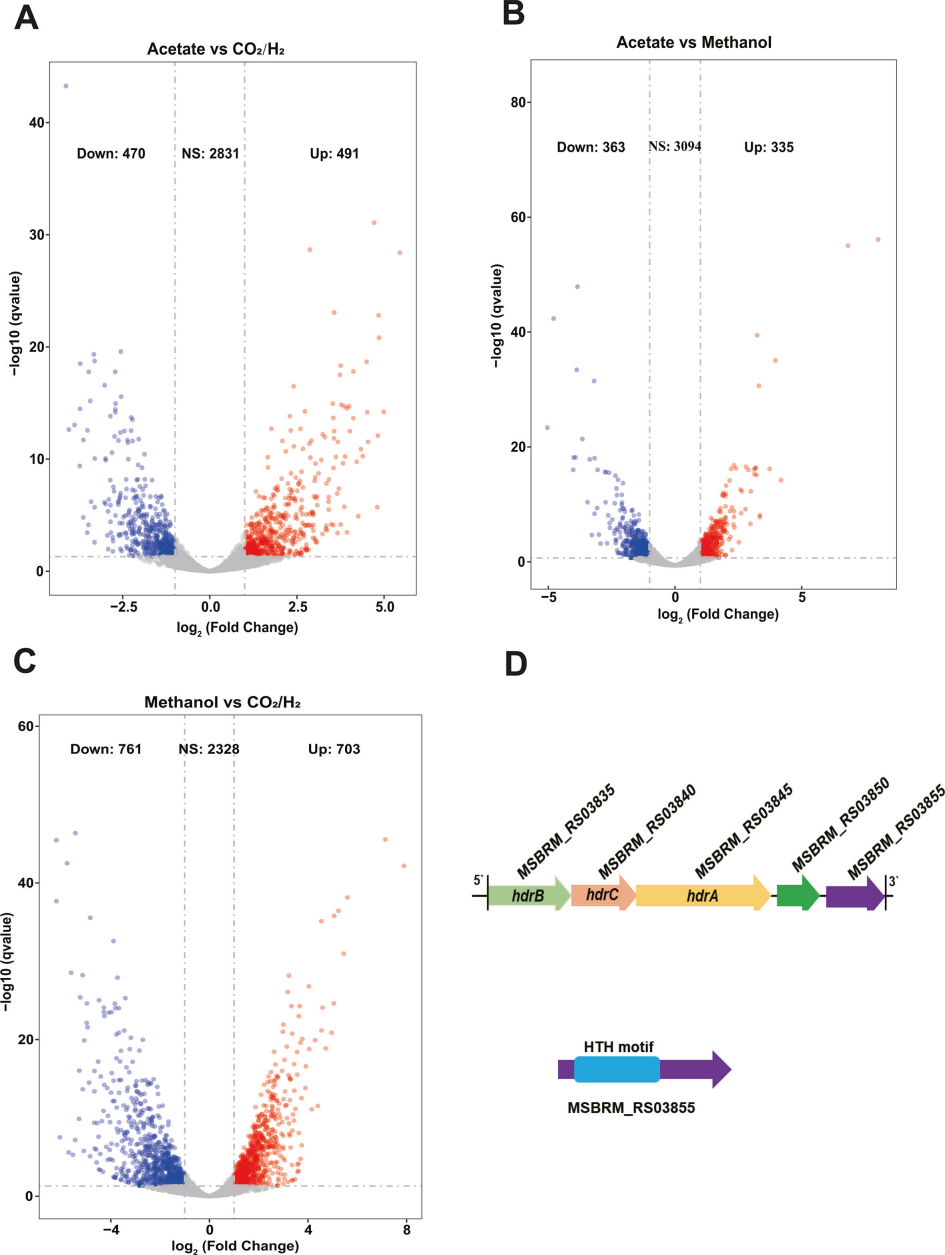
Differential gene transcript abundance for *M. barkeri* cultures grown with H_2_/CO_2_, methanol, or acetate as the substrate. (A) Volcano plot of differentially transcribed genes in the H_2_/CO_2_ substrate culture versus the acetate substrate culture. (B) Volcano plot of differentially transcribed genes in the acetate substrate culture versus the methanol substrate culture. (C) Volcano plot of differentially transcribed genes in the H_2_/CO_2_ substrate culture versus the methanol substrate culture. (D) Arrangement of the *hdrB*, *hdrC*, *hdrA*, MSBRM_RS03850, and MSBRM_RS03855 operons in *M. barkeri*. Volcano plots of differentially transcribed genes in the different substrate cultures versus control. Negative and positive log_2_ fold changes >1 with an adjusted *P* of 0.01 are shown in red and green, respectively. The values marked in red and blue indicate the number of upregulated and downregulated genes, respectively.

Prominent differences were observed in the transcriptome profiles of microorganisms grown under three different substrates. Out of the total 3,792 genes predicted from ORFs, 2,130 genes were found to have nonzero read counts. The CO_2_/H_2_ substrate group showed differential transcription in 25.3% (*n* = 961) of genes relative to the acetate substrate group, with half of these (*n* = 491) being significantly transcribed (Fig. 2A; Fig. S1B). Similarly, the methanol substrate group showed differential transcription in 18.4% (698) of genes relative to the acetate substrate group, with 50% (335/698) of these differentially transcribed genes being upregulated (Fig. 2B). In comparison between the methanol and CO_2_/H_2_ groups, the methanol group showed differential transcription in 38.6% (*n* = 1,464) of genes relative to the CO_2_/H_2_ group, with approximately half (703/1,464) being significantly transcribed. A sizeable share of these genes (55/703) was annotated with an “Energy production and conversion” function (Fig. 2C; Table S2).

Transcriptome data analysis revealed that different substrate groups also differed in the amounts of enriched genes (Fig. S1). The enriched genes observed for *M. barkeri*, when grown under methanol, mapped to the clusters of “Energy production and conversion” (Fig. S1). Among the methanogenic genes, those encoding CO dehydrogenase/acetyl-CoA synthase, acetate kinase, and phosphate acetyltransferase – all involved in the acetoclastic pathway – showed higher transcript abundance in the acetate group than in the other two groups (Table S3). Other genes, such as those encoding methanol corrinoid protein and methanol-cobalamin methyltransferase, exhibited far higher transcript abundance (more than 10-fold) in the methanol group than in the other groups (Table S3). Additionally, the genes encoding heterodisulfide reductase (HdrB, HdrC, and HdrA), which are involved in electron transport systems, showed higher transcript abundance (more than 8-fold) in the CO_2_/H_2_ group than in the other two substrate regimes (Table 2). Interestingly, a gene with an unknown function (MSBRM_RS03855), located downstream of the *hdrBCA* operon, was also significantly transcribed (6–8 folds) in the CO_2_/H_2_ group relative to the methanol and acetate groups (Table 2). The *hdrBCA* and adjacent gene MSBRM_RS03850-MSBRM_RS03855 hint at closely related functions (Fig. 2D; Table 2). According to the annotation of the functional domain, MSBRM_RS03850 and MSBRM_RS03855 were predicted to regulate the *hdrBCA* operon, and may thus be potential regulators of methanogenesis.

**TABLE 2:** Transcription levels of methanogenesis-related heterodisulfide reductase *hdrBCA* genes under three substrate regimes

Gene ID	FPKM^*a*^			Fold change		Protein annotation
	H_2_/CO_2_ (Group 1)	Acetate (Group 2)	Methanol (Group 3)	(Group 1) : (Group 2)	(Group 1) : (Group 3)	
MSBRM_RS03835	427	38	50	11.2	8.5	CoB—CoM heterodisulfide reductase subunit B (HdrB)
MSBRM_RS03840	418	27	43	15.5	9.7	4Fe-4S dicluster domain-containing protein (HdrC)
MSBRM_RS03845	450	41	35	11.0	12.9	FAD-dependent oxidoreductase (HdrA)
MSBRM_RS03850	3	4	2	0.8	1.5	Hypothetical protein
MSBRM_RS03855	24	3	4	8.0	6.0	Hypothetical protein

^*a*^ The reported FPKM values (fragments per kilobase of exon model per million mapped fragments) for each gene represent average values from three replicates of RNAseq analysis, with additional data presented in Tables S3 and S4.

### The MSBRM_RS03855 gene affects growth and CH_4_ production in *M. barkeri* grown under CO_2_/H_2_

**FIG 3:**
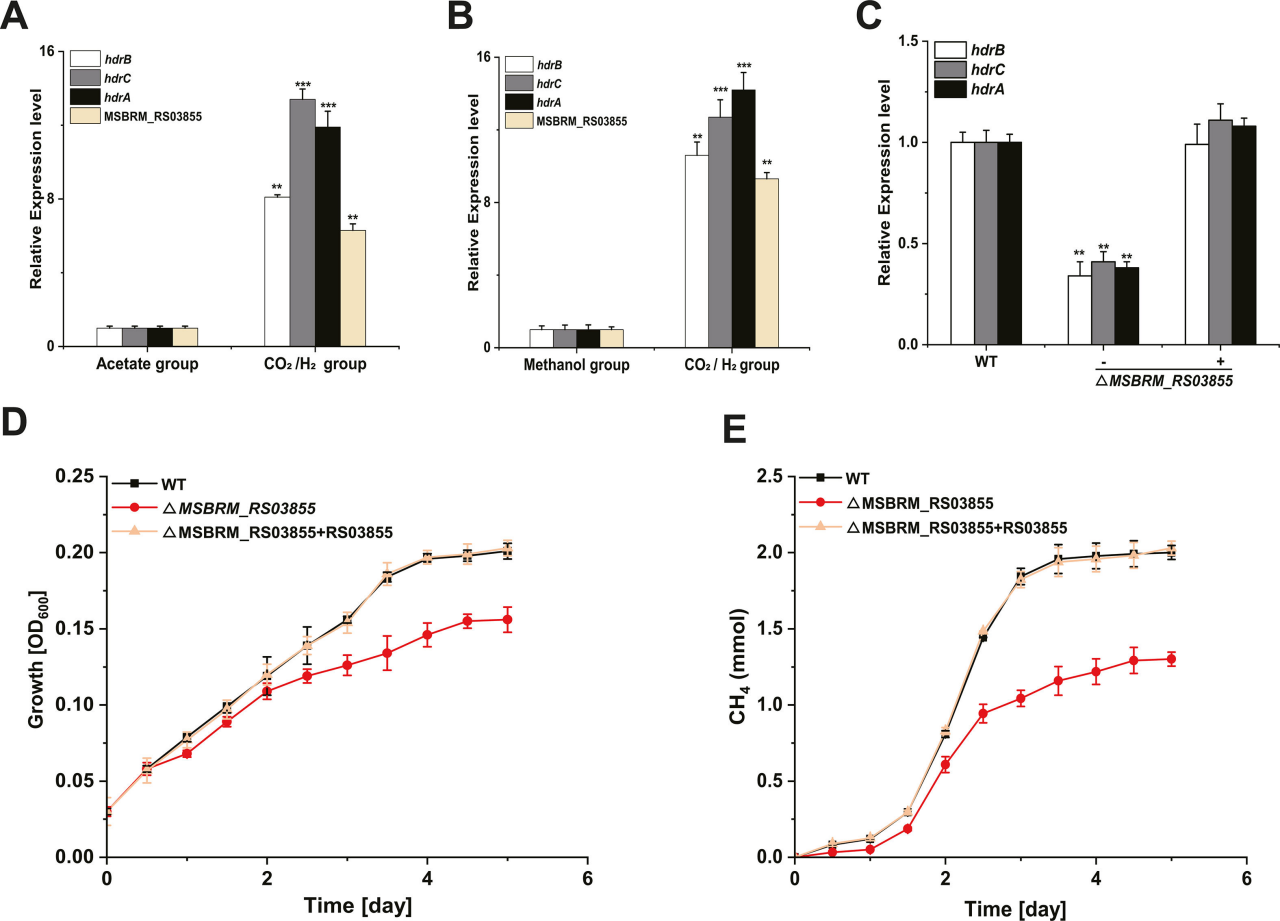
HdrR regulates the transcription of the *hdrBCA* operon. (A) Transcription levels of *hdrB*, *hdrC*, *hdrA,* and *hdrR*, measured by qRT-PCR, in the H_2_/CO_2_ substrate group relative to the acetate substrate group. (B) Transcription levels of *hdrB*, *hdrC*, *hdrA*, and *hdrR*, measured by qRT-PCR, in the H_2_/CO_2_ substrate group relative to the methanol substrate group. (C) Transcription levels of *hdrB*, *hdrC* and *hdrA,* measured by qRT-PCR, in the mutant *M. barkeri* strains relative to wild-type *M. barkeri*. (D) Growth curves for the wild-type *M. barkeri*, as well as the Δ*hdrR* and Δ*hdrR +hdrR* mutants, when grown with H_2_/CO_2_. (E) Accumulation of methane in the headspace of bottles with wild-type *M. barkeri*, as well as the Δ*hdrR*, and Δ*hdrR +hdrR* mutants, when grown with H_2_/CO_2_ over an incubation time of 5 days. In (A), (B), and (C), the values represent the mean value ±SD of three independent experiments; asterisks indicate statistically significant differences between the mutants and wild-type control (***P < 0.001, **P < 0.01), while the lack of an asterisk means that the difference between the mutants and wild-type *M. barkeri* cultures was insignificant.

To gain insights into *hdrBCA* operon transcription, we performed RNA-seq on *M. barkeri* microorganisms grown under different substrate regimes. When the transcriptomes of microorganisms representing the three different substrate regimes were compared, the results indicated that the *hdrBCA* operon has higher transcript abundance (up to 8.5–15.5 fold) when CO_2_/H_2_ served as the substrate. More interestingly, MSBRM_RS03855 (Annotation: hypothetical protein) also showed significant transcript abundance (Table 2). To verify the RNA-seq results, qRT-PCR was performed to measure the mRNA levels of *hdrB*, *hdrC*, *hdrA*, and MSBRM_RS03855 in the different substrate groups; the qRT-PCR results were consistent with the transcriptome profiles (Fig. 3A and B).

To confirm that MSBRM_RS03855 regulates the transcription of the *hdrBCA* operon, targeted ΔMSBRM_RS03855 knock-out *M. barkeri* mutants were created. A complementation ΔMSBRM_RS03855 +RS03855 strain was also established. Furthermore, qRT-PCR was performed to verify the RNA-seq data by measuring the mRNA levels of the *hdrB*, *hdrC*, and *hdrA* genes in the wild-type and mutant strains (Fig. 3C). The results suggest that the inactivation of MSBRM_RS03855 leads to the downregulation of the *hdrBCA* operon transcription. Complementation of MSBRM_RS03855 to the knock-out mutant restored *hdrBCA* operon transcription (Fig. 3C). Growth curves indicated that the deletion of the ΔMSBRM_RS03855 gene affected *M. barkeri* growth when CO2/H2 served as the substrate, while complementation of ΔMSBRM_RS03855 +RS03855 in mutants reversed the observed growth defects (Fig. 3D). Moreover, methane production in the ΔMSBRM_RS03855 strains significantly decreased relative to the wild-type strains, a result which indicates that the deletion of MSBRM_RS03855 negatively affects methanogenesis (Fig. 3E). Growth and CH_4_ production curves indicated that the deletion of the *herR* not affected *M. barkeri* growth and CH_4_ accumulation when methanol or acetate served as the substrate (Fig. S2A through D).

In addition, targeted ΔMSBRM_RS03850 knock-out and complementation ΔMSBRM_RS03850+RS03850 *M*. *barkeri* mutants were constructed. Growth curves indicated that the mutants of the ΔMSBRM_RS03850 and complementation ΔMSBRM_RS03850+RS03850 gene not affect *M. barkeri* growth when CO_2_/H_2_ served as the substrate, compared to wild-type strains (Fig. S2E). As the mutation of MSBRM_RS03855 leads to the downregulation of the *hdrBCA* operon transcription, but MSBRM_RS03850 does not affect *hdrBCA* transcript abundance, this protein may regulate target gene transcription through interactions with the MSBRM_RS03855. To test this hypothesis, independent approaches were used to examine the interaction between these two proteins. A BTH system was used, wherein MSBRM_RS03855 and MSBRM_RS03850 were fused with two fragments of adenylate cyclase (T25 and T18) separately, which restores activity when the fragments are brought into proximity by the interaction of the two chimeric proteins. The results indicated that MSBRM_RS03855 and MSBRM_RS03850 do not directly interact with each other (Fig. S2F).

Overall, these results demonstrate that MSBRM_RS03855 is a regulatory factor of the *hdrBCA* operon, and that its transcription may be important for methanogenesis to properly function (Fig. S3). Collectively, MSBRM_RS03855 is required for the transcriptional regulation of the *hdrBCA* operon; hence, it is renamed as **H**etero**d**isulfide **r**eductase **R**egulator HdrR (MSBRM_RS03855).

### HdrR regulates heterodisulfide reductase (*hdrBCA*) operon transcription

**FIG 4:**
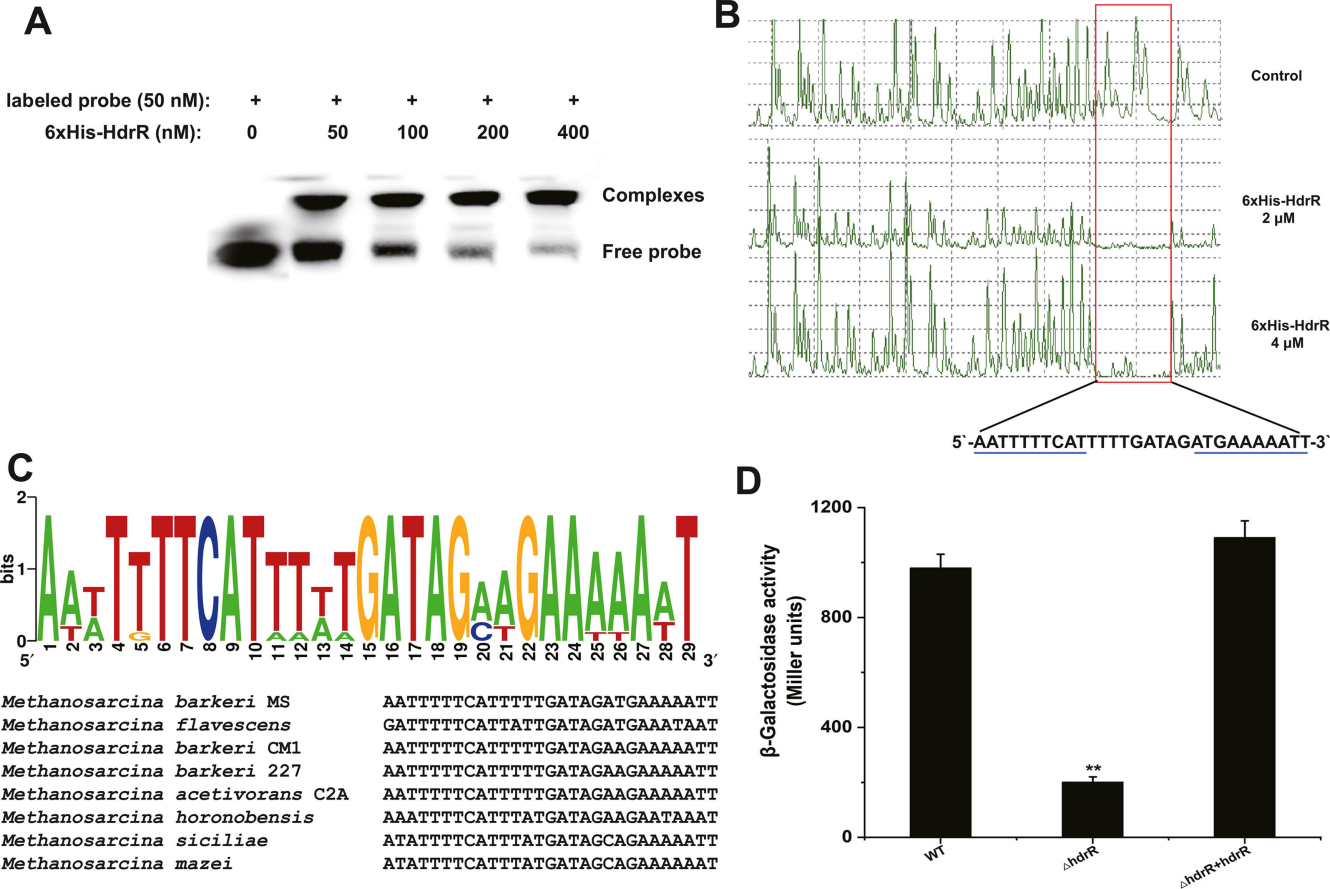
Binding of HdrR to the *hdrBCA* promoter. (A) EMSA analysis of *in vitro* binding of HdrR to the *hdrBCA* operon promoter. Labeled probe, biotin-labeled *hdrBCA* promoter. (B) Identification of the HdrR-protected region in the *hdrBCA* promoter by DNase I footprinting assay and sequence conservation of the identified. (C) HdrR-binding region in different species containing HdrR homologs. (D) The effect of MSBRM_RS03855 on the *hdrBCA* operon promoter, measured by the β-galactosidase activities of wild-type and mutant *M. barkeri* strains with a *P_hdrBCA_ lacZ* transcriptional fusion vector. In (D), the value represents the mean value ± SD for three independent experiments; asterisks indicate statistically significant differences between the wild-type control and mutants (**P < 0.01), while the lack of an asterisk means that the differences between the wild-type and mutant strains were insignificant.

To confirm the regulatory mechanism of HdrR, EMSAs were performed. His-tagged recombinant HdrR protein was purified and incubated with a 111 bp DNA fragment upstream of the *hdrB* start codon. As shown in Fig. 4A, HdrR was able to bind with the DNA fragment and caused a DNA mobility shift. DNase I footprinting analysis identified that the binding region was located on the *hdrBCA* promoter, which is also a conserved motif in *hdrBCA* promoter sequences (Fig. 4B). Sequence analysis of the upstream region of the *hdrBCA* operon from different archaea species that contain HdrR detected obvious palindromic sequences (Fig. 4B and C). These results provide evidence that HdrR directly acts on the promoter of the *hdrBCA* operon. To confirm the regulatory roles of HdrR in connection with the promoter of the *hdrBCA* operon, we created a *lacZ* reporter for the *hdrBCA* promoter in the wild-type, Δ*hdrR* mutant and Δ*hdrR+hdrR* complementation mutant strains. In agreement with the qRT-PCR results, the β-galactosidase assays indicated that the *hdrBCA* promoter is positively controlled by the *hdrR* protein (Fig. 4D). The above results demonstrate that HdrR binds the *hdrBCA* promoter.

### The helix-turn-helix domain of HdrR is likely involved in DNA binding

**FIG 5:**
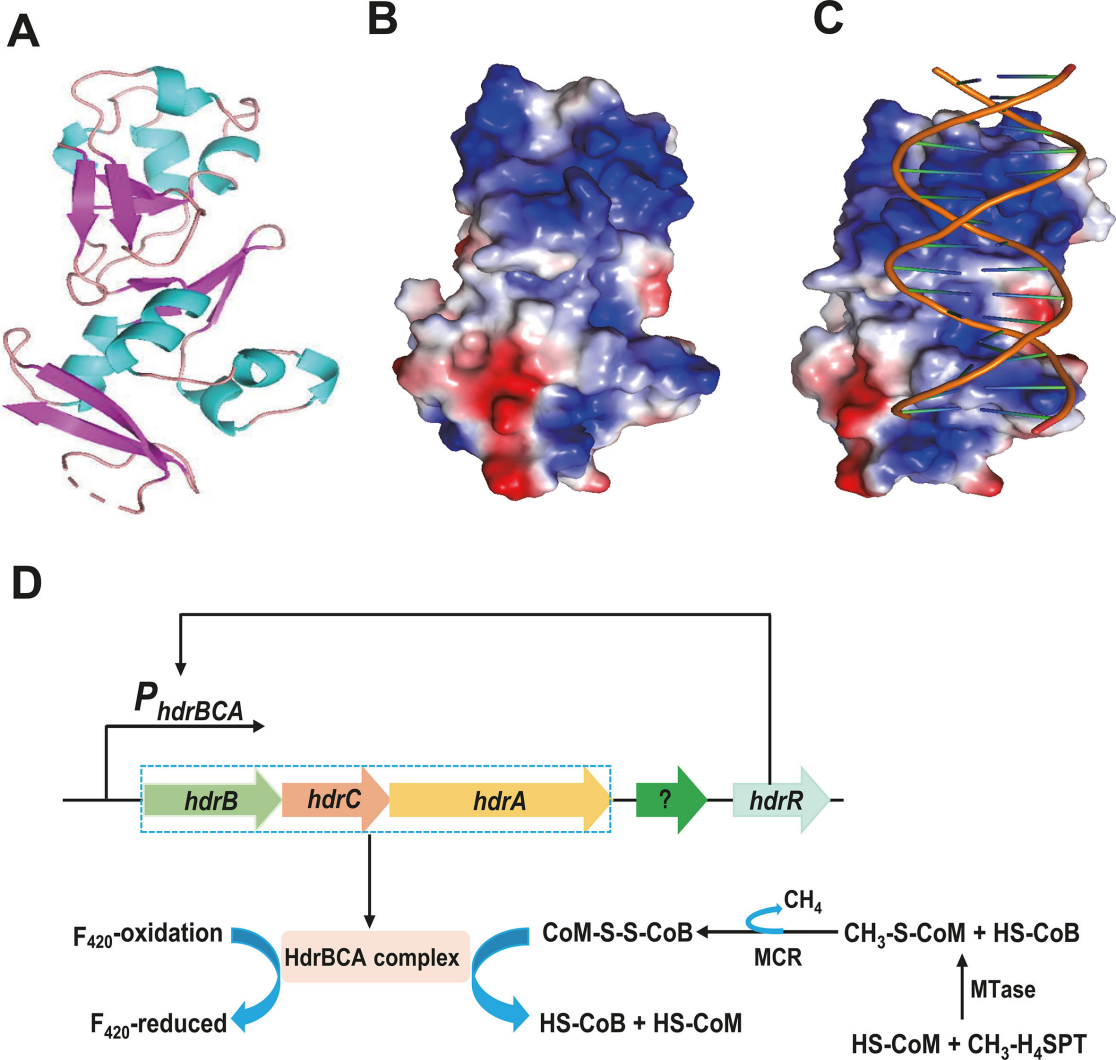
AlphaFold2 produces highly accurate representations of the HdrR structure. (A) An overview of HdrR model structure. (B) A surface-electrostatic view of HdrR, which highlights the continuous and strongly positive charges on one side of the molecule. (C) An illustration of the *hdrBCA* promoter docked to HdrR created using AutoDock VinA. (D) Schematic diagram of how HdrR regulates the transcription of the *hdrBCA* operon.

In order to understand how HdrR, as a transcription factor, binds to the *hdrBCA* operon promoter, the protein’s structure was constructed using AlphaFold2 (Fig. 5A). The structure of HdrR includes a winged HTH domain at the N-terminus, a feature commonly observed in DNA-binding proteins. To gain further insight into the structural details of the HdrR-binding domain, target DNA sequences were docked to HdrR using AutoDock VinA. As shown in Fig. 5B, an extended surface patch with continuous and strongly positive charges was identified on one side of the molecule, representing an appropriate ligand-binding site for DNA engagement (Fig. 5C). Further structural model studies of HdrR revealed certain characteristics of winged HTH motifs, which are likely involved in DNA binding as well. Taken together, HdrR serves as a model to reveal how certain regulatory factors control the expression of key enzymes in the methanogenic pathway (Fig. 5D).

## DISCUSSION

It is well known that the gene transcription of methanogenic metabolic pathways *M. barkeri* needs to be spatially and temporally regulated. However, the gene transcription regulation of the methanogenic heterodisulfide reductase *hdrBCA* operon has been far less studied. Here, we report a novel regulator, HdrR, which exclusively activates transcription of the heterodisulfide reductase operon *hdrBCA* in *M. barkeri*, all of which are essential for methanogenesis. Further structural model analyses revealed a helix-turn-helix domain, which is likely involved in DNA binding. These findings demonstrate that HdrR is essential for the proper transcription of *hdrBCA* and is important for efficient methanogenesis of *M. barkeri*. This HdrR may be similar to classical two-component system regulators, but we have not yet found the upstream sensing regulator. What signals may trigger these regulators, particularly unknown sense regulator, to activate the methanogenic heterodisulfide reductase *hdrBCA* operon transcription? This is an interesting question, the answer to which may uncover new signaling pathways or links between the methanogenic pathway and other known cellular processes.

In summary, we provide novel insight into the mechanisms regulating *hdrBCA* operon transcription in the model methanogen *M. barkeri*. This clarified that HdrR serves as a regulator of heterodisulfide reductase *hdrBCA* operon transcription during methanogenesis, which expands our understanding of the unique regulatory mechanisms that govern methanogenesis. These findings revealed how genetic expression can regulate methanogenesis in methanogen, this knowledge is important, as it can be later used to develop a green and low-carbon future for people.

## Data Availability

Raw sequencing reads and processed data have been deposited in SRA under accession number PRJNA1105475. All other data are available in the article or the supplemental material.

## SUPPLEMENTAL MATERIAL

Supplemental figures aem.00691-24-s0001.pdf Figures S1 to S5.

Supplemental legends aem.00691-24-s0002.pdf Legends for supplemental figures and tables.

Supplemental tables aem.00691-24-s0003.xlsx Tables S1 to S8.

ASM does not own the copyrights to Supplemental Material that may be linked to, or accessed through, an article. The authors have granted ASM a non-exclusive, world-wide license to publish the Supplemental Material files. Please contact the corresponding author directly for reuse.
