# Supplementary material for: The novel regulator HdrR controls the transcription of the heterodisulfide reductase operon hdrBCA in Methanosarcina barkeri
Source: Appl Environ Microbiol. 2024 May 29;90(6):e00691-24. doi: 10.1128/aem.00691-24 (PMC11218639; doi:10.1128/aem.00691-24)
Supplement: Supplemental figures — Figures S1 to S5. [file aem.00691-24-s0001.pdf]

FIG. S1

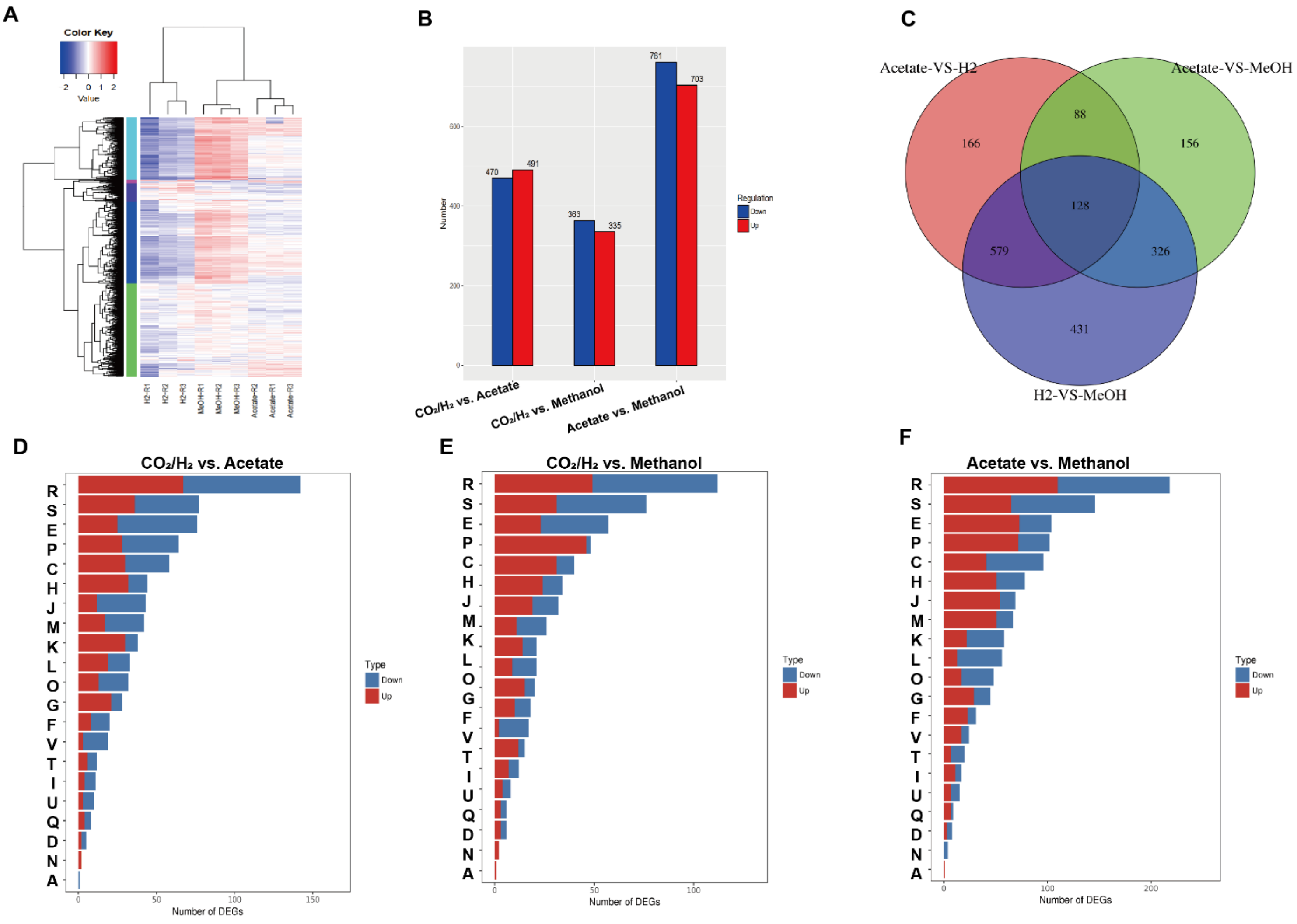
A
Color Key
-2 0 1 2
Value
H2-R1
H2-R2
H2-R3
MOH-R1
MeOH-R2
MOH-R3
Acetate-R2
Acetate-R1
Acetate-R3
B
Number
0
200
400
600
470
491
363
335
761
703
Regulation
Down
Up
CO₂/H₂ vs. Acetate
CO₂/H₂ vs. Methanol
Acetate vs. Methanol
C
Acetate-VS-H2
Acetate-VS-MeOH
H2-VS-MeOH
166
88
156
128
579
326
431
D
CO₂/H₂ vs. Acetate
R S E P C H J M K L O G F V T I U Q D N A
Type
Down
Up
0 50 100 150
Number of DEGs
E
CO₂/H₂ vs. Methanol
R S E P C H J M K L O G F V T I U Q D N A
Type
Down
Up
0 50 100
Number of DEGs
F
Acetate vs. Methanol
R S E P C H J M K L O G F V T I U Q D N A
Type
Down
Up
0 100 200
Number of DEGs

FIG. S2

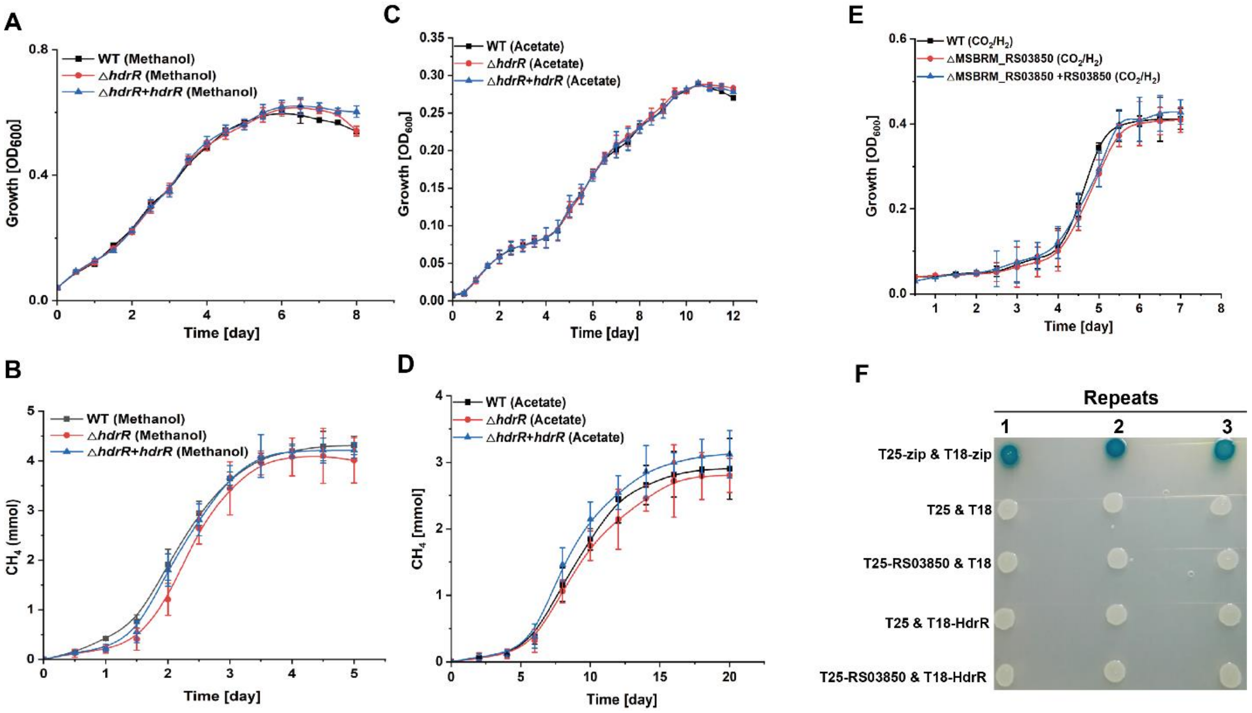
A
WT (Methanol)
△hdrR (Methanol)
△hdrR+hdrR (Methanol)
Growth [OD600]
Time [day]
B
WT (Methanol)
△hdrR (Methanol)
△hdrR+hdrR (Methanol)
CH4 (mmol)
Time [day]
C
WT (Acetate)
△hdrR (Acetate)
△hdrR+hdrR (Acetate)
Growth [OD600]
Time [day]
D
WT (Acetate)
△hdrR (Acetate)
△hdrR+hdrR (Acetate)
CH4 [mmol]
Time [day]
E
WT (CO2/H2)
△MSBRM_RS03850 (CO2/H2)
△MSBRM_RS03850 +RS03850 (CO2/H2)
Growth [OD600]
Time [day]
F
Repeats
1
2
3
T25-zip & T18-zip
T25 & T18
T25-RS03850 & T18
T25 & T18-HdrR
T25-RS03850 & T18-HdrR

FIG. S3

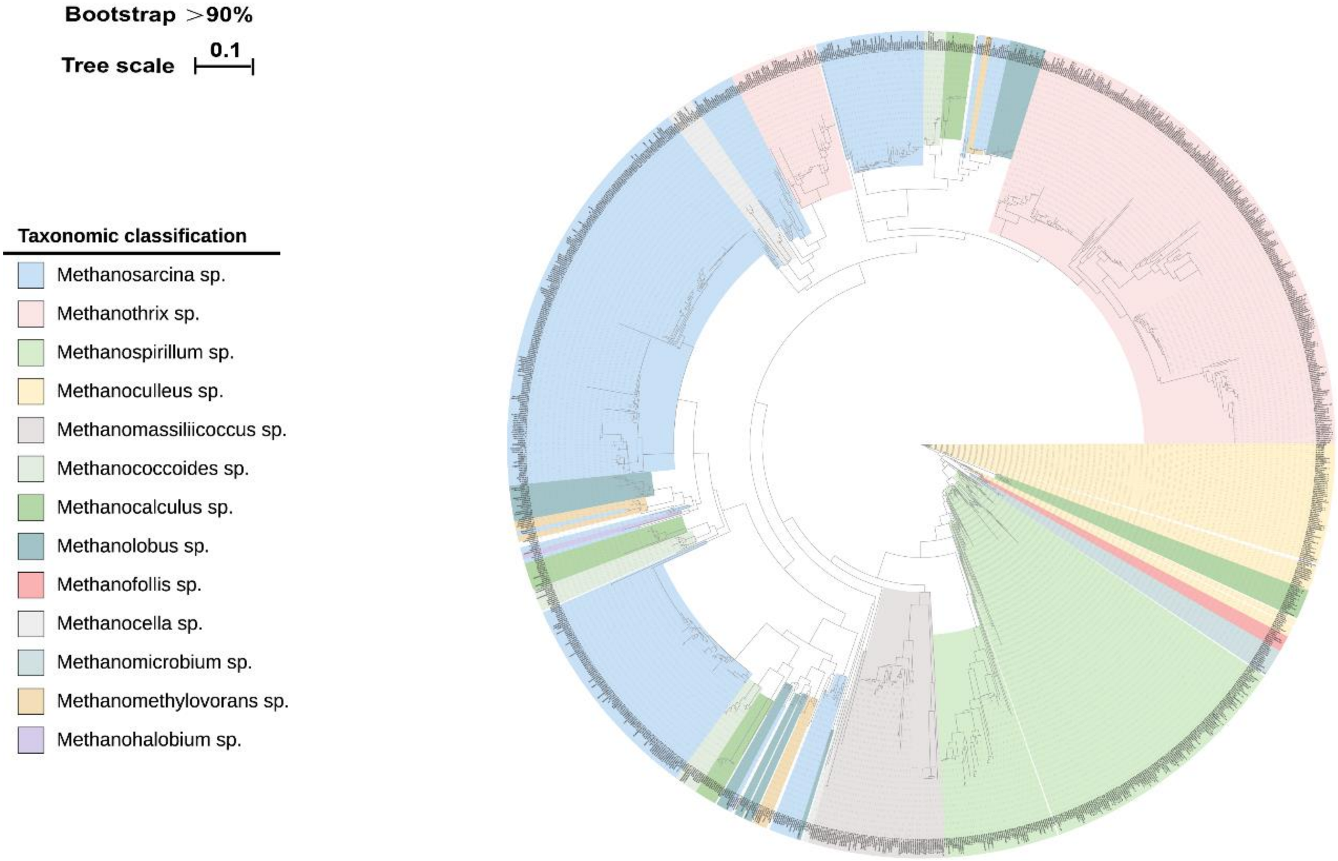
Bootstrap >90%
Tree scale 0.1
Taxonomic classification
Methanosarcina sp.
Methanothrix sp.
Methanospirillum sp.
Methanoculleus sp.
Methanomassiliicoccus sp.
Methanococcoides sp.
Methanocalculus sp.
Methanolobus sp.
Methanofollis sp.
Methanocella sp.
Methanomicrobium sp.
Methanomethylovorans sp.
Methanohalobium sp.

FIG. S4

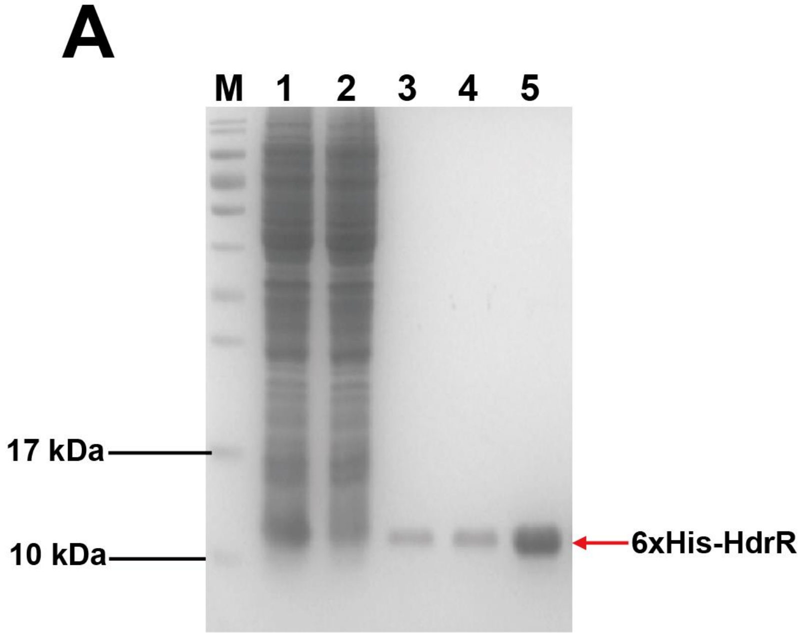

FIG. S5

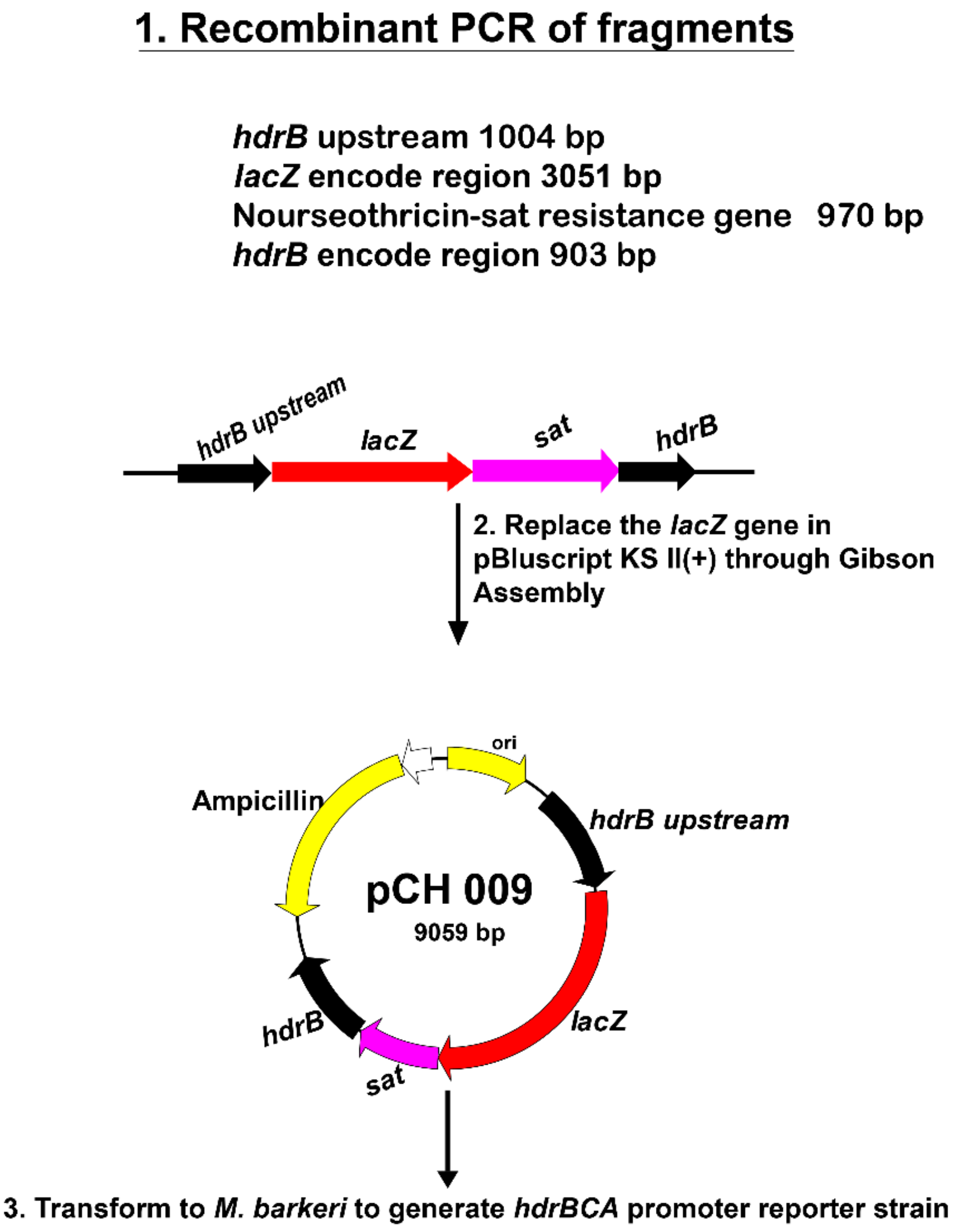
1. Recombinant PCR of fragments
hdrB upstream 1004 bp
lacZ encode region 3051 bp
Nourseothricin-sat resistance gene 970 bp
hdrB encode region 903 bp
hdrB upstream
lacZ
sat
hdrB
2. Replace the lacZ gene in pBluscript KS II(+) through Gibson Assembly
ori
Ampicillin
hdrB upstream
pCH 009
9059 bp
lacZ
sat
hdrB
3. Transform to M. barkeri to generate hdrBCA promoter reporter strain
