# Supplementary material for: The novel regulator HdrR controls the transcription of the heterodisulfide reductase operon hdrBCA in Methanosarcina barkeri
Source: Appl Environ Microbiol. 2024 May 29;90(6):e00691-24. doi: 10.1128/aem.00691-24 (PMC11218639; doi:10.1128/aem.00691-24)
Supplement: Supplemental legends — Legends for supplemental figures and tables. [file aem.00691-24-s0002.pdf]

Supplementary Figure legends

FIG. S1. Comparisons of gene transcript abundance across different substrate regimes. (**A**) Hierarchical clustering analysis of differentially transcribed genes in cultures of M. barkeri grown in H2/CO2, acetate, or methanol; (**B**) Pairwise comparisons (adjusted $p<0.01$) of gene transcript abundance across different treatments; (**C**) Gene co - transcription analysis; (**D-F**) The enriched genes were selected based on the pairwise comparison (adjusted $p<0.01$) results of different treatments, and are annotated based on the arCOG database. All data are presented as mean values.

FIG. S2. **The *hdrR* gene does not affect growth and CH_4 production in *M. barkeri* grown under methanol or acetate.** (**A**) Growth curves for the wild-type *M. barkeri*, as well as the △*hdrR* and △*hdrR* + *hdrR* mutants, when grown with methanol; (**B**) Accumulation of methane in the headspace of bottles with wild-type *M. barkeri*, as well as the △*hdrR* and △*hdrR* + *hdrR* mutants, when grown with methanol over an incubation time of 5 days. (**C**) Growth curves for the wild-type *M. barkeri*, as well as the △*hdrR* and △*hdrR* + *hdrR* mutants, when grown with acetate. (**D**) Accumulation of methane in the headspace of bottles with wild-type *M. barkeri*, as well as the △*hdrR* and △*hdrR* + *hdrR* mutants, when grown with methanol over an incubation time of 20 days. (**E**) Growth curves for the wild-type *M. barkeri*, as well as the △MSBRM_RS03850 and △MSBRM_RS03850 + RS03850 mutants, when grown with H_2/CO_2. (**F**) BTH analysis of the interaction between MSBRM_RS03850 and HdrR in *vivo*. In (**A**), (**B**), (**C**), (**D**), and (**E**) the values represent the mean value ± SD of three independent experiments.

FIG. S3. Phylogenetic affiliation of HdrR/HdrR-homologous amino acid sequences present in archaea. Phylogenetic tree based on HdrR/HdrR-homologous amino acid sequence using a maximum likelihood algorithm. The scale bar indicates the number of amino acid substitutions per site.

FIG. S4. Purification of the recombinant 6xHis-HdrR protein. M, protein molecular marker; Lane 1, *E. coli* BL21 containing the pET28a plasmid (negative control); Lane 2, 6xHis-HdrR synthesis was induced by the pET28a-*hdrR* vector, after which the protein was further purified; Lane 3, protein solubilized in His-bind binding buffer; Lane 4, protein solubilized in His-bind

washing buffer; Lane 5, protein solubilized in His-bind elution buffer.

FIG. S5. Sequence map for the pCH004 plasmid. A diagram showing how the pCH004 plasmid was cloned and used to construct a β-gal reporter strain for *hdrBCA* promoter activity assays in *M. barkeri*.

Supplementary Tables

Table S1. Functional statistics of enriched genes annotated based on the arCOG database.

Table S2. The proportion of functional categories in which each of the enriched genes was involved.

Table S3. The transcript abundance of methanogenic genes under three distinct substrate treatments.

Table S4. The transcript abundance of key non-methanogenic genes under three distinct substrate treatments.

Table S5. Information on all the selected genomes (8028 in total) was downloaded from the NCBI genome database.

Table S6. Summary of HdrR homologs in archaea genomes.

Table S7. List of the primer sequences used in this study.

Table S8. List of the microorganism strains and plasmids used in this study.
